# Factors Associated with Farmers’ Participation in the U.S. Conservation Reserve Program (CRP)

**DOI:** 10.1007/s00267-026-02600-3

**Published:** 2026-08-24

**Authors:** Suraj Upadhaya, Shreesha Pandeya, Lisa A. Schulte

**Affiliations:** 1 School of Agriculture and Natural Resources; College of Agriculture, Health, and Natural Resources, Kentucky State University, Frankfort, KY 40601 USA; 2 School of Forest, Fisheries, and Geomatics Sciences, Institute of Food and Agricultural Sciences, University of Florida, Gainesville, FL 32603 USA; 3 Department of Natural Resource Ecology and Management, Iowa State University, Ames, IA 50011 USA; 4 Bioeconomy Institute, Iowa State University, Ames, IA 50011 USA

**Keywords:** Agriculture, Conservation adoption, Conservation policy, Farm bill, Ecosystem services, Private lands, Working lands conservation

## Abstract

The Conservation Reserve Program (CRP), the largest U.S. private lands conservation initiative, incentivizes farmers to retire environmentally sensitive land to reduce erosion, improve water quality, and enhance wildlife habitat. The program is currently under consideration for reauthorization by Congress. We assessed social, economic, and ecological factors associated with CRP enrollment to evaluate program effectiveness and guide future policy. Analysis of a 5-year (2015–2019) survey of Iowa farmers revealed several factors positively associated with participation. Most strongly, the farm-level ecological variable of the mean percent slope was positively associated with CRP adoption. A one-unit increase in mean percent slope increases the odds of enrollment by threefold. Farmers who received technical assistance from non-governmental agencies for the implementation of conservation practices, a social variable, were 88% more associated with enrollment in CRP. Cropland area, an economic variable, increased the odds of enrolling in CRP by 47%. Several additional variables were positively associated with CRP enrollment, including receipt of additional cost-share, attitudes toward nutrient management, involvement in watershed management groups, agronomic capacity, education, access to information from public sources, and age. The amount of land in pasture and the proportion of farmland rented were negatively associated with farmers’ CRP enrollment. Our findings highlight current program strengths that enhance conservation outcomes as well as potential future adjustments to further address cost and trust barriers.

## Introduction

The Conservation Reserve Program (CRP) is the largest private lands conservation program in the United States. It incentivizes farmers to take environmentally sensitive land out of commodity crop production and establish perennial cover for multiple conservation benefits important to society now and in the long term. While having a large total annual expenditure of $1.9 billion (USDA FSA, 2023), CRP has achieved positive soil, water, wildlife, and other environmental outcomes (Bigelow et al., 2020; USDA FSA, 2021; USDA FSA, 2024; Vandever et al., 2023). For example, CRP water quality practices enrolled in fiscal year (FY) 2021 are estimated to have prevented the loss of 161 million kgs of nitrogen, 32 million kgs of phosphorus, and 136 million metric tons of sediment loss, and to have mitigated more than 12 million metric tons of carbon dioxide equivalent (CO2e) (USDA FSA, 2023). Hansen (2007) evaluated the environmental outcomes of the CRP and found that the program reduces total annual erosion by 222 to 248 million tons, equivalent to an 11–12% reduction from the level at the time of analysis.

Farm Bills have become the principal conduit for federal investment in soil, water, and habitat protection on private lands (McGranahan et al., 2013). CRP was originally established by the 1985 Farm Bill and is periodically reauthorized as part of an omnibus bill (McGranahan et al., 2013). It is administered by the U.S. Department of Agriculture Farm Service Agency (FSA). In exchange for a yearly rental payment, farmers enrolled in the program agree to remove land from crop production and plant species that will improve environmental health and quality (Brorsen et al., 1996). Contracts for land enrolled in CRP range from 10 to 15 years in length. For farmland owners seeking to enroll in the Conservation Reserve Program (CRP), eligibility requires that they have owned the land for at least one year and that the land has been planted with an agricultural commodity crop for at least four of the previous six years. The Grassland Conservation Reserve Program (GCRP), which constitutes the largest and qualitatively distinct component of CRP, applies specifically to grasslands most at risk of conversion to cropland and follows different enrollment criteria designed to preserve working grassland ecosystems. Landowners may be additionally eligible for cost-share assistance to establish conservation practices, including tree planting, grassland restoration, or wetland development. The 2018 U.S. Farm Bill, which authorizes the CRP, was extended by the American Relief Act in late 2024 through September 30, 2025, and remains the governing law while Congress negotiates a new Farm Bill for 2026 and beyond.

**Fig. 1:**
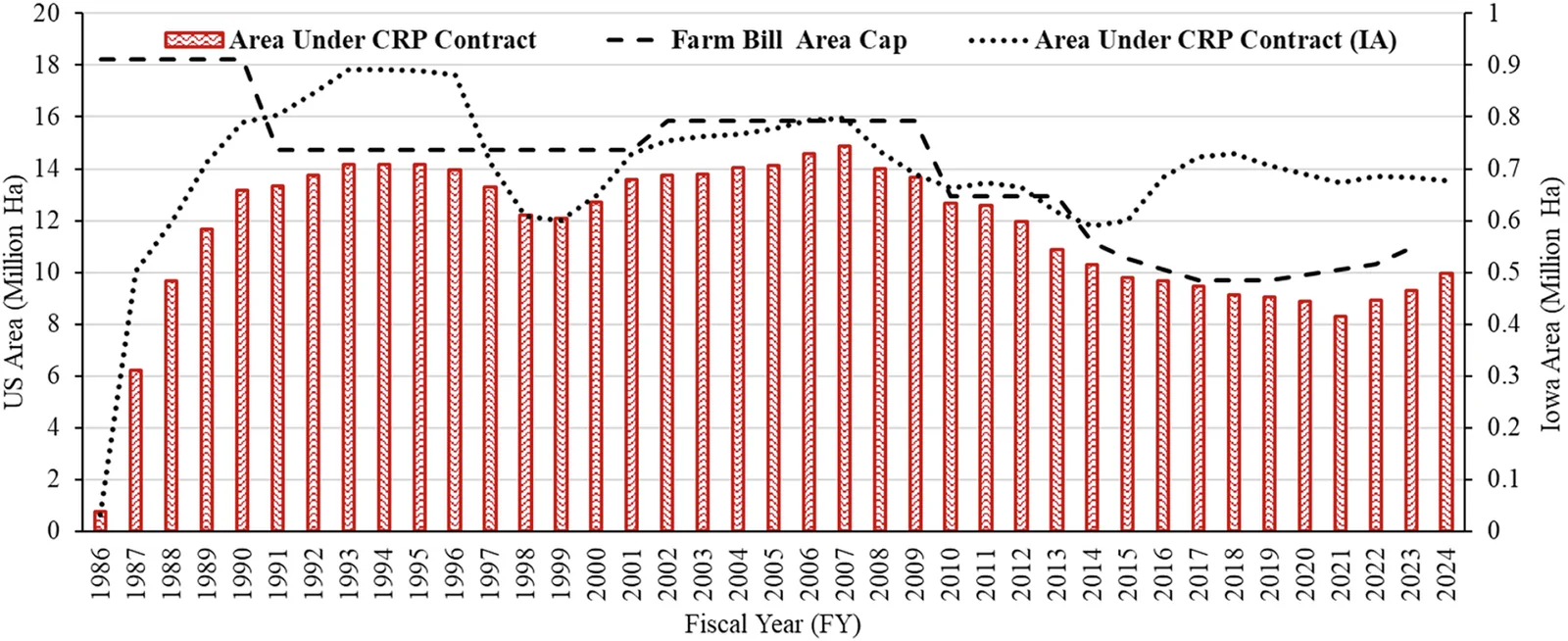
United States Department of Agriculture Conservation Reserve Program (CRP) enrollment cap and total area enrolled over time for the U.S. and Iowa (IA) (USDA FSA, 2024)

Despite numerous benefits, land enrollment in CRP peaked at 14.9 million hectares in 2007 and has consistently not met its cap for over a decade (USDA FSA, 2021, 2024). In the 2018 Farm Bill, the U.S. Congress called for an incremental increase in enrollment limits in CRP. For fiscal year (FY) 2019, CRP enrollment was capped at 9.7 million hectares: 9.9 million hectares for FY 2020, 10.1 million hectares in FY 2021, 10.3 million hectares in FY 2022, and 11 million hectares in FY 2023. Enrollment at the end of 2023 was 10 million hectares, about 1.0 million hectares below the cap (Fig. 1).

Unmet enrollment occurs against a backdrop of agricultural land-use change (Morefield et al., 2016). Substantial portions of expiring CRP land were converted to row crop production in the 2010s. Morefield et al. (2016) reported that over 30% (approximately 530,000 hectares) of CRP land in a 12-state Midwestern region transitioned to row crops between 2010 and 2013, with the highest conversions occurring in southern Iowa, the Dakotas, and Nebraska. Similarly, Bigelow et al. (2020) found that nearly 79% of 1.9 million hectares of CRP land exiting the program between 2013 and 2016 were converted to row-crop cultivation. More recent national evidence indicates that these dynamics coexist with substantial gross cropland abandonment. The recent paper by Xie et al. (2024) mapped cropland abandoned across the conterminous U.S. between 1986 and 2018, with only about 19.6% of abandoned lands enrolled in CRP by 2020, underscoring ongoing competition among production, conservation, and alternative land uses. Regardless, the economic, environmental, and ecological impacts of not meeting CRP’s cap may be substantial and are likely to have profound implications for soil, water, and wildlife resources in the U.S. (Allen and Vandever, 2012; Bigelow et al., 2020; Morefield et al., 2016; Vandever et al., 2023).

The success of CRP is predicated on farmers’ voluntary participation. High commodity crop prices, which encourage landowners to put even low-yielding land into row-crop production, are known to strongly affect enrollment (Stubbs, 2014; Thapa et al., 2024). Prior research on private landowners’ participation in voluntary conservation initiatives indicates the influence of multiple interacting factors (Baumgart-Getz et al., 2012; Houser et al., 2024; McCann and Claassen, 2016; Prokopy et al., 2019, 2008; Reimer and Prokopy, 2014; Thapa et al., 2024; Upadhaya et al., 2023; Upadhaya and Arbuckle, 2021). Socio-economic factors such as education, age, farm size, gender, capital, income, access to information, and conservation awareness and attitudes have been identified to influence the adoption of conservation practices (Hendricks and Er, 2018; Lark et al., 2022, 2015; Sullins et al., 2021).

Given CRP’s status as the largest private-lands conservation program in the U.S., a comprehensive understanding of the social, economic, and ecological factors can elucidate the facilitators and barriers to enrollment. To date, however, we know of no multidimensional studies that assess the factors associated with farmers’ enrollment in CRP. Such understanding could enable program improvements, especially as the U.S. Congress considers renewal legislation.

We assessed social, economic, and ecological factors associated with CRP enrollment by farmers in the state of Iowa with data from a 2015–2019 survey. Iowa is a highly agricultural state in the central portion of the U.S. Corn Belt. Approximately 83% of the state is in agricultural land uses, where it accounted for over 17% of the nation’s corn production and more than 12% of its soybean yield, making it the leading producer of both crops (USDA NASS, 2023). It is also among the top producers of eggs, hogs, cattle, ethanol, and agriculturally based bioproducts in the United States (USDA NASS, 2023). As of November 2024, Iowa ranked sixth among states in terms of CRP contract signups, with 53,888 farms enrolling 678,120 ha, and had the highest total annual rental payout at $405,338,000 (USDA FSA, 2024). The CRP enrollment patterns in Iowa closely follow national trends (Fig. 1), supporting the representativeness of the Iowa data in illustrating broader program dynamics. Understanding factors influencing farmers’ enrollment in CRP is essential for enhancing program design and implementation, ensuring continued effectiveness in achieving conservation goals.

## Methods

### Data Collection

Data were from a survey of Iowa farmers conducted over five years to support the implementation of the Iowa Nutrient Reduction Strategy (INRS), a science-based framework that aims to reduce nutrient loads in waterways that lead to the Gulf of America/Mexico (INRS, 2020). The survey focused on farmers’ knowledge, attitudes, and behaviors related to using soil and water conservation practices to reduce nutrient loss (Nowatzke and Arbuckle, 2018). Between 2015 and 2019, the survey was sent to 14,139 farmers and stratified by hydrologic unit code six (HUC6) watersheds. A total of 6,006 farmers completed the survey, resulting in a 42% response rate. The sample targeted row crop producers operating at least 60 hectares, who, while comprising roughly 50% of all Iowa farmers, manage about 90% of the state’s farmland (USDA NASS 2024). As such, the survey provides strong coverage of the population most relevant to nutrient management and conservation efforts promoted by the INRS.

### Data Analysis

Farmer enrollment in CRP was assessed through a binary variable based on their response to a survey question, “How much of the land is enrolled in CRP?” Farmers who reported CRP enrollment were coded as “1”; otherwise, “0”. We included 28 different farmer-reported personal- and farm-level data, described in the following sections, as explanatory variables in a binomial regression model describing farmer CRP enrollment. The survey year was also included as a control variable. We analyzed data using the Statistical Package for Social Sciences (SPSS-IBM) software, version 22, and R software (R Core Team, 2023). We used a binary logistic regression model, with enrollment in CRP as the binary dependent variable (Hardle and Simar, 2014). Given the number of predictors in our model and the sample size, we used the Benjamini-Hochberg (BH) procedure to control false discovery rates (Benjamini and Hochberg, 1995). This adjustment was necessary to minimize the risk of false associations arising from multiple hypothesis tests. We report both uncorrected and BH-adjusted p-values in Table 2. We also assessed multicollinearity using variance inflation factors (VIF) and evaluated model fit. Variables with only low VIFs were included in the final model. After BH correction, to assess the practical significance of the remaining predictors, we calculated predicted probabilities of CRP enrollment for representative farmer profiles using the fitted logistic regression model. For each profile, variables of interest were set to specific values (typically the 25th or 75th percentile) while all other variables were held at their sample mean, and the model-implied probability of enrollment was computed. This allowed us to identify combinations of characteristics at which the predicted enrollment probability exceeds 50%.

### Farmer and Farm Characteristics

We incorporated several variables representing farmer and farm characteristics that are commonly used in research on conservation adoption (Prokopy et al., 2019): age, gender, education cropland area (*Crop.Acre*), pasture area (*Pasture.Acre*), percent rented cropland (*Per.Rented.Crop*), gross farm sales (*GFS*), and whether any of the cropland owned by the farmers was adjacent to or connected to water bodies such as creeks, streams, rivers, or lakes (*Water.Border*) (SP1). We included a farm-level measure of vulnerability to erosion, average percent slope (*Slope.Mean*), using the 30-m elevation data prepared by the USDA/NRCS National Geospatial Center of Excellence, because it has been found to be a positive farm-level predictor of conservation behavior (Morton et al., 2015; Prokopy et al., 2019). We further included the binary measures of whether or not farmers have a nutrient management plan (*Nut.Mgmt.Plan*) and raise livestock, as they have been shown to be associated with the use of some conservation practices (Lu et al., 2022).

### Attitudes and Awareness

Farmers’ awareness of and attitudes toward conservation practices are consistent predictors of adoption (Prokopy et al., 2019). To assess the impact of awareness and attitudes on the adoption of conservation practices, we measured farmers’ awareness of the INRS and their attitudes toward it. The attitudes variable was derived from four items measuring farmers’ attitudes towards the INRS, which were ranked on a five-point agreement scale ranging from not at all knowledgeable (1) to very knowledgeable (5) (SP1).

### Perceived Capacity

Perceived behavioral control is an individual’s beliefs about their capability to make choices about their behaviors within a given situation (Fishbein and Ajzen, 2010). It is generally assumed to lead to more positive conservation behavior (Arbuckle and Roesch-McNally, 2015; Lee et al., 2018; Reimer et al., 2012). Farmers’ perceived capacity to implement different practices for water quality improvement in Iowa was assessed through a set of questions that asked survey respondents to assess seven potential barriers to water quality improvement based on a five-point scale ranging from strongly disagree (1) to strongly agree (5). We created two summated scales to represent farmers’ perceived economic (“*Capacity.Econ*”) and agronomic capacity (“*Capacity.Agron*”) (SP1).

### Information Sources

Research consistently indicates that the source of information available to farmers is a significant predictor of their soil and water conservation behavior (Carlisle, 2016; Prokopy et al., 2019). Awareness campaigns that disseminate information about water conservation issues and potential solutions can also play a crucial role in fostering collective action among farmers (Davenport and Seekamp, 2013). In this study, we wanted to identify the sources of information farmers used to learn about the different conservation practices. The survey provided farmers with eight options, which included three private-sector sources (seed company representative, agricultural retailer, and independent/private crop adviser or agronomist), three public-sector sources (Iowa State University Extension and Outreach, Natural Resources Conservation Service, and another government agency), and two mass media sources (farm press and popular press). We then created four information source variables based on the farmers’ responses, resulting in a four-point count variable (0-3) for the private and public sources (*Info.Priv* and *Info.Pub*, respectively) and binary variables (0-1) for the farm and popular press sources (*Farm.Press* and *Pop.Press*, respectively) (SP1).

### Influential Actors

We aimed to identify the influence of various agricultural stakeholders on farmers’ conservation decisions because different agricultural actors have different degrees of influence (Prokopy et al., 2019). For instance, Lee et al. (2018) found that those farmers who were influenced by private sector actors were less likely, and those influenced by public sector entities were more likely, to adopt cover crops. We asked farmers to rate 14 agricultural stakeholders on a scale of 1 (no influence) to 5 (very strong influence). Using factor analysis, we grouped these 14 stakeholders into four categories: public sector, private sector, family/farmers/landlords, and organizations that sponsor on-farm research and demonstrations. We then generated scales by summing the responses for each group and dividing them by the number of items in that group. The public sector stakeholders (*Infl.Pub*) included Iowa State University Extension, Iowa Department of Agriculture and Land Stewardship, United States Department of Agriculture Natural Resources Conservation Service (USDA NRCS) or county Soil and Water Conservation District, and Iowa Water Quality Initiative (WQI). Seed companies, custom operators/applicators, local agricultural retailers (e.g., fertilizer, agricultural chemical dealers, co-op), and independent/private crop advisers/agronomists comprised private sector entities (*Infl.Priv*). The family/farmers/landlords group (*Infl.Fam*) included family members, other farmers, and the landlord/farm management firm (SP2). The on-farm research and demonstration groups (*Infl.On-farm*) included Iowa Learning Farms, Practical Farmers of Iowa, and the Iowa Soybean Association.

### Relational Networks

Involvement in conservation networks is an important factor contributing to farmers’ engagement in conservation behaviors (Lu et al., 2022; Prokopy et al., 2019). By connecting with conservation-related agencies and organizations, farmers can increase their capacity to adopt pro-environmental behaviors both individually and collaboratively (Davenport and Seekamp, 2013). To assess farmers’ contact with conservation-related organizations and agencies, we created a binary variable Cost Share (*CS*) from responses to the following question: “In the last five years, have you received cost-share to help you fund conservation practices?” The second variable, Technical Assistance from Government Agency (*TA.G*), was constructed from the response to the following question: “In the last five years, have you received conservation technical assistance from a governmental agency (Soil and Water Conservation District or NRCS/FSA)?” The third variable, Technical Assistance from Non-Governmental Organizations (TA.NG), was constructed from the responses to the following question: “In the last five years, have you received conservation technical assistance from a non-governmental organization?” Farmers’ “no” responses were assigned a “0” and “yes” a “1”.

Involvement in organized watershed management activities has been found to positively affect soil and water quality outcomes (Church and Prokopy, 2017; Upadhaya and Arbuckle, 2021). We thus included farmer responses to a question asking if they were engaged in any watershed management groups in the watershed where their farm operations were located (*Inv.W.Group*). Farmers who responded “yes” were assigned a value of 1, and 0 for no.

## Results

Out of the 6006 farmers surveyed, incomplete responses on some variables reduced the effective sample size to 3151. Of the 3151 survey responses analyzed, 33.4% of farmer respondents indicated that they were enrolled in CRP. The logistic regression model was statistically significant (χ² = 1865.4, *p* < 0.001) and explained 63% (Nagelkerke R²) of the variation in farmers’ enrollment in CRP (Table 2). Among the 28 variables analyzed, 13 remained statistically significant predictors after applying a Benjamini-Hochberg correction for multiple testing (*p* < 0.05) (Table 2), suggesting that our binary logistic model accounted for most of the variation in the dependent variable. Among the 28 total variables analyzed, six of the 15 social, five of the eight economic, and two of the four ecological variables were statistically significant predictors at a *p* < 0.05 (Table 2).

Thirteen ecological, social, and economic explanatory variables were significant positive predictors of CRP enrollment (Table 2). Most strongly, the farm-level ecological variable of the mean percent slope was positively associated with CRP adoption. A one-unit increase in mean percent slope increases the odds of enrollment by about threefold. Farmers who received technical assistance from non-government agencies (*TA.NG*) for conservation practices, a social variable, were 88% more likely to enroll in CRP. Farmers who received cost-share (*C.S*.) to help with conservation practices were 65% more likely to enroll in CRP than those who did not.

Farm size (*Crop.Acre*), an economic variable, was also positively associated. A one-unit increase in crop area (ha) increased the odds of enrolling in CRP by 47%. Farms with CRP were 350 ha in size on average compared to 299 ha for those without. A positive attitude toward nutrient retention was positive and significant, with an odds ratio indicating that a one-unit increase in the 5-point attitude scale corresponds to a 47% increase in the odds of CRP enrollment. Farmers involved in watershed management groups (*Invl.Wat.Mgmt.Group*) were 42% more likely to enroll in CRP than those who were not. The perceived lack of agronomic capacity (*Capacity.Agron*) was significant and positively associated with a one-unit increase on the scale, corresponding to a 29% increase in the odds of enrollment. Farmer education had a strong, positive effect, with an increase in education by one unit increasing the odds of enrollment by 18%. On average, farmers who enroll in CRP have a bachelor’s degree, while farmers who do not enroll in CRP are not college-educated. One of the four information channels through which farmers learned about nutrient retention was significant: learning about nutrient retention through public sources (*Info.Public*) had a positive effect, with a one-unit increase in the number of public sector sources associated with a 17% increase in the odds of enrollment. Farmer age had only a minor effect, with an increase in age by one year increasing the odds of CRP enrollment by 3%.

Conversely, two economic variables, the extent of pastureland (*Pasture.Acre*) and rented cropland (*Per.Rented.Crop*), were significantly negatively associated with enrollment in CRP, though not strongly so. Farmers with more pasture were less likely to enroll, with an 18% decrease in odds for a one-unit increase in pasture acres. Similarly, an increase in the percent of rented cropland by 1 unit decreased the odds of enrollment by 1%. On average, landowners who enrolled in CRP rented a lower proportion of the land they farmed (37.6%) compared to farmers who did not enroll in CRP (53.1%).

Fifteen variables we evaluated were not significant predictors of CRP enrollment. Notably, gross sales, livestock ownership, bordering water, and perceived economic capacity were not significant, nor were any variables related to social influence. Gender was not a significant predictor, but our sample included a few non-male respondents (Table 1).

**Table 1:** Descriptive statistics of social (soc), economic (econ), and ecological (ecol) variables associated with farmer data from the Iowa Nutrient Reduction Strategy Survey, 2015–2019

Variable	Enrolled in CRP (1)		Not Enrolled in CRP (0)	
**Farmer Characteristics**	Min/Max	Mean/SD	Min/Min	Mean/SD
Age (soc)	26/89	60.19/10.88	20/98	56.88/12.49
Gender (soc) ^a^	0/1	0.97/0.16	0/1	0.98/0.13
Education (soc) ^b^	1/4	2.09/0.98	1/4	2.02/0.96
**Farm Characteristics**				
Total Crop Ha (econ)	0/5698	350.05/389.3	0/4168.3	299.06/315.71
Total Pasture Ha (econ)	0/809.37	22.21/51.79	0/809.37	22.09/55.03
Percent Crop Rented (econ)	0/100	37.6/33.90	0/100	53.1/37.92
Gross Sales (econ) ^c^	1/8	5.21/1.89	1/8	4.97/1.79
Mean Percent Slope (ecol)	3/14.19	5.56/1.69	0.1/11.26	2.65/1.74
Water Border (ecol)	0/1	0.88/0.32	0/1	0.77/0.42
Nutrient Management Plan (ecol)	0/1	0.34/0.47	0/1	0.27/0.44
Livestock (ecol)	0/1	0.38/0.48	0/1	0.41/0.49
**Attitudes and Awareness**				
Attitude (soc)	1/5	3.68/0.50	1/5	3.55/0.52
Awareness (soc)	1/5	3.04/0.98	1/5	2.89/0.96
**Perceived Capacity**				
Economic Barriers (econ)	1/5	3.32/0.75	1/5	3.42/0.75
Agronomic Barriers (econ)	1/5	2.82/0.69	1/5	2.83/0.68
**Information Sources**				
Information (Private) (soc)	0/3	0.62/0.94	0/3	0.59/0.91
Information (Public) (soc)	0/3	1.8/1.08	0/3	1.63/1.14
Farm Press (soc)	0/1	0.83/0.38	0/1	0.8/0.42
Popular Press (soc)	0/1	0.5/0.50	0/1	0.51/0.50
**Influential Actors**				
Influence (Public) (soc)	1/5	2.57/0.89	1/5	2.37/0.93
Influence (Private) (soc)	1/5	2.07/0.83	1/5	2.11/0.84
Influence (On-Farm Research) (soc)	1/5	1.7/0.81	1/5	1.69/0.84
Influence (Family) (soc)	1/5	2.3/0.87	1/5	2.31/0.91
**Relational Networks**				
Technical Assistance (Government) (econ)	0/1	0.7/0.46	0/1	0.46/0.5
Technical Assistance (Non-Government) (soc)	0/1	0.16/0.36	0/1	0.08/0.27
Cost Share (econ)	0/1	0.64/0.48	0/1	0.35/0.48
Involvement in Watershed Management Group(soc)^d^	0/1	0.36/0.48	0/1	0.25/0.44

^a^0:female; 1:male

^b^1:High School or Less; 2:Some College; 3:Bachelor's; 4: Grad School/Prof Degree

^**c**^None=1; <$50 K = 2; $50K-$150 K = 3; $150K-$250 K = 4; $250K-350K = 5; $350K-500K = 6; $500K-$1000 K = 7;  > $1000 K = 8

^d^0:no; 1 = yes

**Fig. 2:**
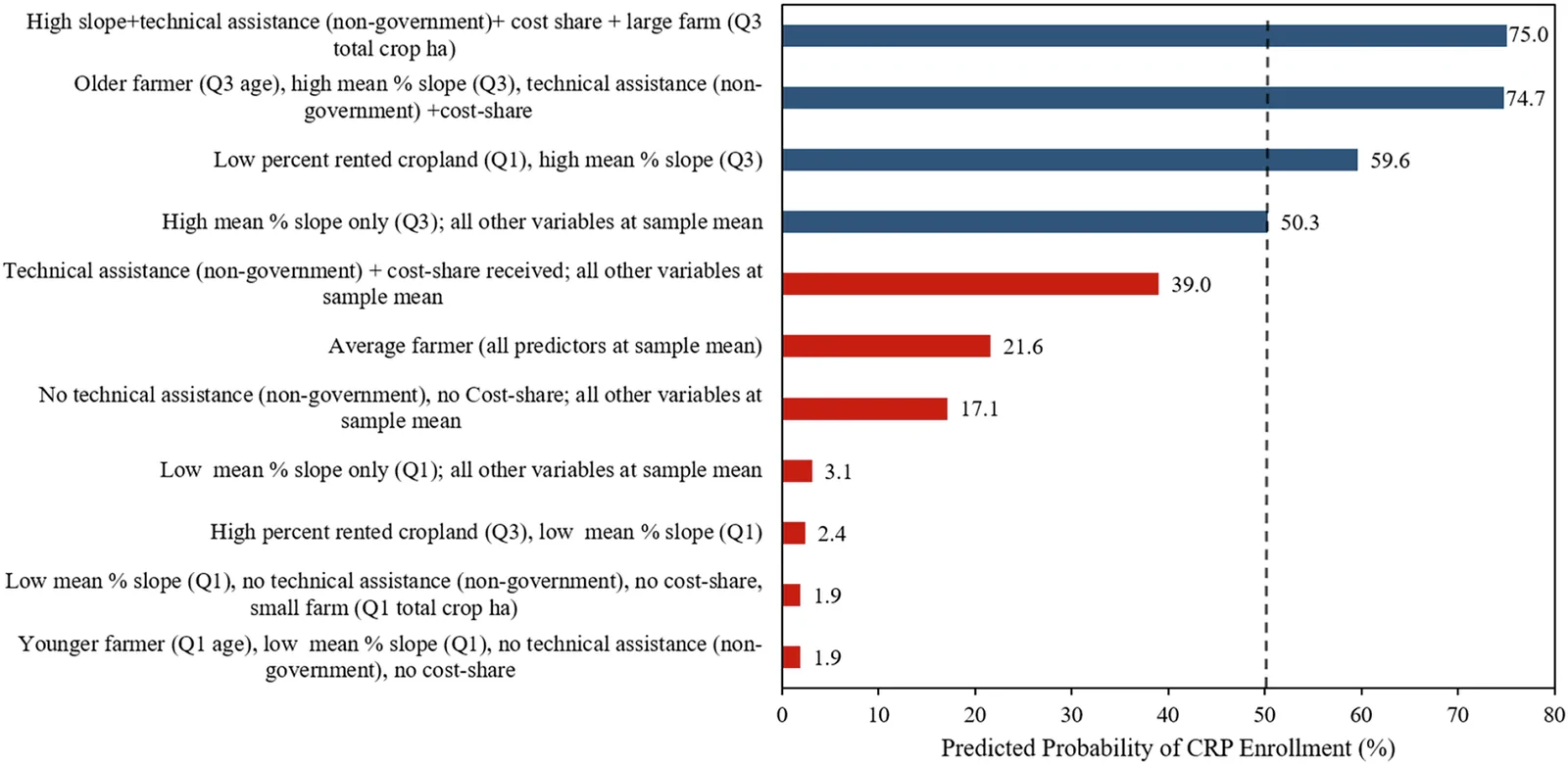
Predicted probability of Conservation Reserve Program (CRP) enrollment for representative farmer profiles, derived from the binomial logistic regression model (Table 2). Q1 and Q3 denote the 25th and 75th percentiles of the sample distribution for that variable. The dashed vertical line marks the 50% threshold, above which a farmer profile is more likely than not to enroll in CRP. Bars in blue indicate profiles at or above this threshold; bars in red indicate profiles below it

To better understand the practical significance of these associations beyond relative odds ratios, we calculated predicted probabilities of CRP enrollment for representative farmer profiles, holding all other variables at their sample means (Fig. 2). A farmer with average characteristics across all predictors had a 21.6% predicted probability of enrollment in CRP. This is well below the threshold (50%) at which enrollment becomes more likely than not. Mean percent slope was the single strongest driver of practical significance (Table 2): a farmer at the 75th percentile (Q3) of mean slope alone had a predicted enrollment probability of 50.3%, crossing this threshold independent of any other factor. By contrast, receiving both technical assistance from a non-government organization and cost-share support raised the predicted probability to only 39.0% when other characteristics were held at the mean, indicating that these social and economic supports, while statistically significant, are not independently sufficient to make enrollment the modal outcome. When favorable land characteristics (high mean % slope, larger cropland area) were combined with technical assistance from non-governmental organizations and cost-share support, the predicted probability rose to 75.0%. Conversely, a farmer with a low mean % slope, no technical assistance from non-governmental organizations, no cost-share, and a smaller farm size had a predicted probability of just 1.9% of enrolling in CRP (Fig. 2).

**Table 2:** Binomial logistic regression of social, economic, and ecological variables on Iowa farmers’ enrollment in the U.S. Conservation Reserve Program (CRP), with Benjamini-Hochberg false discovery rate correction

Variables and Model Descriptors	B	SE	Z	OR	95% CI	*P*	P(BH)
(Intercept)	−303.7	98.742	−3.076				
Year	0.145	0.049	2.961	1.156	1.050, 1.273	0.003^**^	0.009^**^
**Farmer Characteristics**							
Age (soc)	0.029	0.005	5.489	1.029	1.019, 1.039	<0.001^***^	<0.001^**^
Gender (soc)	−0.141	0.366	−0.385	0.868	0.423, 1.781	0.700	0.784
Education (soc)	0.169	0.060	2.835	1.185	1.054, 1.332	0.005^**^	0.011^**^
**Farm Characteristics**							
Total Crop Ha (log) *Crop.Acre* (econ)	0.387	0.098	3.956	1.473	1.216, 1.784	<0.001^***^	<0.001^***^
Total Pasture Ha (log) *Pasture.Acre* (econ)	−0.200	0.031	−6.384	0.819	0.770, 0.871	<0.001^***^	<0.001^***^
Percent Crop Rented *Per.Rented.Crop* (econ)	−0.008	0.002	−4.613	0.992	0.989, 0.995	<0.001^***^	<0.001^***^
Gross Sales *GFS* (econ)	−0.024	0.046	−0.511	0.977	0.892, 1.069	0.610	0.718
Mean Percent Slope *Slope.Mean* (ecol)	1.082	0.043	25.385	2.952	2.715, 3.209	<0.001^***^	<0.001^***^
Water Border *Water.Border* (ecol)	0.084	0.158	0.527	1.087	0.797, 1.483	0.598	0.718
Nutrient Mgmt Plan *Nut.Mgmt.Plan* (ecol)	0.140	0.124	1.124	1.150	0.901, 1.467	0.261	0.406
Livestock (ecol)	−0.149	0.137	−1.086	0.862	0.658, 1.127	0.278	0.409
**Attitudes and Efficacy**							
Attitude (soc)	0.390	0.120	3.263	1.477	1.169, 1.868	<0.001^***^	<0.001^***^
Awareness (soc)	−0.015	0.069	−0.220	0.985	0.860, 1.128	0.826	0.856
**Perceived Capacity**							
Economic Barriers *Capacity.Econ* (econ)	−0.052	0.076	−0.679	0.950	0.818, 1.102	0.497	0.663
Agronomic Barriers *Capacity.Agron* (econ)	0.255	0.085	2.995	1.290	1.092, 1.524	0.003^**^	0.009^**^
**Information Sources**							
Information (Private) *Info.Priv* (soc)	0.011	0.067	0.160	1.011	0.886, 1.153	0.873	0.873
Information (Public) *Info.Pub* (soc)	0.156	0.059	2.664	1.169	1.042, 1.311	0.008^**^	0.017^*^
Farm Press *Info.Press* (soc)	0.215	0.161	1.335	1.240	0.904, 1.702	0.182	0.335
Popular Press *Pop.Press* (soc)	−0.150	0.117	−1.287	0.860	0.684, 1.082	0.198	0.335
**Influential Actors**							
Influence Public *Infl.Pub* (soc)	0.027	0.090	0.303	1.028	0.862, 1.226	0.762	0.821
Influence (Private) *Infl.Priv* (soc)	−0.132	0.083	−1.602	0.876	0.745, 1.030	0.109	0.218
Influence (On−Farm Research) *Infl.On*−*Farm* (soc)	−0.114	0.090	−1.272	0.892	0.749, 1.064	0.203	0.335
Influence (Family) *Infl.Fam* (soc)	0.052	0.075	0.699	1.054	0.910, 1.220	0.484	0.663
**Relational Networks**							
Technical Assistance (Gov) *TA.G* (econ)	0.073	0.146	0.502	1.076	0.808, 1.433	0.616	0.718
Technical Assistance (Non−Gov) *TA.NG* (soc)	0.633	0.178	3.565	1.884	1.330, 2.668	<0.001^***^	0.002^**^
Cost Share *CS* (econ)	0.498	0.140	3.563	1.645	1.251, 2.163	<0.001^***^	0.002^**^
Involvement in Watershed Management Group *Inv.W.Group* (soc)	0.352	0.125	2.811	1.421	1.112, 1.816	0.005^**^	0.011^**^
**Model Descriptors**							
Nagelkerke Pseudo R^2^	63%						
Correct Prediction %	84.3%						
N	3151						
CRP Enrolled	0.334						
Non Enrolled	0.666						
Model x2	1865.384***						
Log	1071.34						
Multiple testing correction	Benjamini Hochberg FDR (28 predictors)						

*p ≤ 0.05; **p ≤ 0.01; ***p ≤ 0.001

## Discussion and Conclusions

The U.S. Congress is currently considering legislation to reauthorize CRP and thereby affect conservation outcomes on millions of hectares of private lands in the U.S. The economic and conservation benefits of the program are clear: it provides farmers with a supplemental income source for fields that need to lie fallow or are only marginally productive, stabilizes soil and improves water quality, and offers tangible benefits for outdoor enthusiasts. For instance, by restoring marginal farmland to grassland, wetland, or native-vegetation cover under the Conservation Reserve Program, the resulting habitat boosts populations of game species (e.g., waterfowl, upland birds, deer) and non-game wildlife, which creates better hunting, fishing, birdwatching, and wildlife-viewing opportunities (Bigelow et al., 2020; Hansen, 2007; USDA FSA, 2021; Vandever et al., 2023). Also, cleaner water and healthier aquatic and riparian ecosystems improve conditions for anglers and paddlers, enhancing the quality of nature-based recreation.

Support for CRP is variable among agricultural stakeholder groups (Kadam et al., 2025), however, and enrollment is substantially lower than its 2007 peak (Fig. 1). This variation is due to differences in motivations, economic considerations, and perceived benefits. Some farmers and landowners are motivated by financial incentives and conservation goals, while others view the program as conflicting with production priorities, particularly when commodity prices are high or land is perceived as more profitable in active production (Stuart and Gillon, 2013; Thapa et al., 2024). Additionally, stakeholder alignment with CRP goals is influenced by community norms, risk perceptions, and trust in government programs, which affects willingness to enroll or re-enroll in CRP (Reddy et al., 2020; Sullins et al., 2021). Our study highlights the multifaceted factors associated with farmers’ decisions to enroll in CRP. The significant ecological, social, and economic variables we identified provide insights into the complexity of enrollment behavior and suggest targeted policy and outreach strategies. Our analysis should be interpreted as descriptive and associational rather than causal. The survey data allows us to identify farm, farmer, and network characteristics that are statistically associated with CRP enrollment, but they do not allow us to determine whether changing any one factor would cause farmers to enroll. This distinction is important for policy interpretation. Causal estimates would be needed to evaluate whether specific program design changes, such as expanding technical assistance or modifying payment structures, would increase participation. By contrast, the associations identified here are most useful for understanding how current CRP features align with particular farm characteristics and for identifying producer groups, landscapes, or outreach channels that may warrant closer attention.

Our study also supports previous research demonstrating that ecological outcomes, such as soil and nutrient retention, are critical motivators for landowners to engage in conservation practices (Gelfand et al., 2011; Otto et al., 2018). Mean percent slope on farmland, an ecological factor, was highly correlated with CRP enrollment in our study (Table 2, Fig. 2). The strong association between mean percent slope and CRP enrollment should not be interpreted as evidence that slope independently causes participation. Rather, slope likely captures a broader set of land-capability and program-eligibility considerations. Sloped cropland may be more erosion-prone, less profitable in annual row-crop production, and more likely to benefit from perennial cover or conservation practices such as grassed waterways, contour buffers, prairie strips, or no-till systems. Thus, the mean % slope result is consistent with CRP’s role in targeting environmentally sensitive or marginal cropland. However, slope may also be a proxy for unmeasured factors, including expected yield, erosion history, highly erodible land status, rental-rate attractiveness, or farmer perceptions of production risk. Future research linking survey responses to parcel-level CRP records and qualitative farmer interviews would help clarify these mechanisms. Farmers' perceived agronomic capacity was also positively associated with their likelihood of participating in the program. CRP’s role in improving soil health, reducing erosion, and enhancing water quality provides substantial environmental incentives for farmers. This result also speaks to CRP’s success in transitioning environmentally sensitive lands from commodity crop production.

Economic considerations are important in farmers’ decision-making processes regarding CRP enrollment (Bigelow et al., 2020; Thapa et al., 2024). Our study found that farm size, receipt of cost-share assistance, and higher perceived agronomic capacity positively influenced CRP participation. Larger farms with more resources are better positioned to take advantage of CRP’s financial incentives, which can offset potential income losses from agricultural production (Bigelow et al., 2020). Larger farms tend to have more total acreage, which gives them greater flexibility: they can enroll a portion of their land in CRP while still retaining enough acreage in production to absorb yield risk and maintain farm income, especially when considering risk management and crop insurance costs (Khanal et al., 2025).

Our results point toward more targeted, rather than universal, policy interventions. Our predicted probability results (Fig. 2) indicate that these social and economic levers are most effective when targeted at farms that are already land-marginal, rather than applied broadly across the farming population. Mean percent slope was the single strongest driver of predicted enrollment likelihood, with farms at the 75th percentile of slope alone exceeding the 50% threshold of predicted enrollment. By contrast, technical assistance from non-governmental organizations and cost-share support alone, without favorable land characteristics, raised the predicted probability to only 39%, well below the point at which enrollment becomes the more likely outcome. This suggests that policy interventions are likely to be most cost-effective when concentrated on farms with steep or otherwise marginal land, where program support can convert already-favorable land conditions into actual enrollment decisions, rather than when distributed uniformly across farms regardless of land quality. In practice, these points suggest prioritizing outreach, technical assistance, and cost-share allocation for farms identified through existing slope and soil-quality data (e.g., NRCS Web Soil Survey) as having a high proportion of marginal or highly erodible land, rather than broad-based educational or incentive campaigns aimed at the general farming population. Taken together, these findings suggest that advisory support and financial incentives are most likely to translate into enrollment when paired with, rather than substituted for, favorable land conditions.

While landowners have decision authority over their lands under the voluntary conservation framework for private lands in the U.S., social factors play a crucial role in CRP enrollment. Farmers engaged in watershed management groups were significantly more likely to enroll in the CRP, highlighting the importance of community-based conservation initiatives. This finding aligns with previous research emphasizing the role of social networks and community engagement in conservation behavior (Davenport and Seekamp, 2013; Prokopy et al., 2019, 2008). Public sector information sources also positively influenced enrollment, suggesting that extension services and government-led educational programs are effective in promoting conservation behaviors (Lee et al., 2018). Although geospatial information on slope and proximity to waterbodies is technically available through public platforms (e.g., NRCS Web Soil Survey and state GIS portals), our findings indicate that farmers who received guidance from non-government conservation advisors were significantly more likely to enroll in CRP. This suggests that the limiting factor is not data availability per se, but rather the translation of that information into locally relevant, trusted, and actionable recommendations, which NGOs and watershed councils are well-positioned to provide (Lee et al., 2018).

Conversely, rented cropland and a higher proportion of pasture acres were negatively associated with CRP enrollment. This suggests that many farmers may view renting cropland or maintaining pasture as providing not only financial returns but also other benefits such as flexibility in land use, support for existing livestock systems, and preservation of family or community farming traditions, which they perceive as more valuable than CRP participation, or they are less likely to be eligible and/or garner less financial assistance. This is especially true during periods of high commodity prices, where market forces encourage farmers to put their CRP land back into production (Adjei et al., 2024; Stubbs, 2014). Research showed that landowners are more likely to retain grass cover after their CRP contracts end when they have positive program experiences and are motivated by aesthetics or wildlife benefits, whereas strong financial motives tend to reduce such enrollment and persistence (Barnes et al., 2023).

Interactions among ecological, economic, and social factors create complex decision-making settings for farmers. Our research indicates that CRP addresses many of the interconnected influences that affect farmer enrollment. With Congress revisiting the Farm Bill, our findings point to several program strengths and also opportunities to amplify conservation benefits, including:

- Ranking offers by ecological leverage factors such as slope and proximity to water, as currently performed through the Environmental Benefits Index, channels new contracts toward the lands that deliver the greatest soil- and nutrient-retention benefits and improve farmer profitability.
- Pairing rental payments with signup incentive payments (SIPs) and practice incentive payments (PIPs) and providing technical assistance, as is already done, are important facilitators of enrollment. Additional grants to support watershed-level peer learning could further address the cost, knowledge, and trust barriers our model identified.
- Introducing shorter “stepping-stone” contracts, perhaps with streamlined alignment with the Natural Resources Conservation Service (NRCS) Environmental Quality Incentives Program (EQIP) or Conservation Stewardship Program (CSP), and allowing flexible mid-term practice tweaks, such as modifying vegetation mixes or adding complementary buffers without penalty, would ease opportunity-cost concerns that surface when commodity prices spike.
- Similarly, eliminating the 25% payment reduction penalty for periodic non-emergency haying and grazing could ease opportunity-cost concerns, a change supported by our finding that farms with greater pasture acreage were significantly less likely to enroll in CRP. Restrictions and financial penalties on forage use reduce the program’s appeal for operators who depend on haying and grazing systems.
- Reserving a portion of new acreage for smaller or marginal farms could broaden participation. Our results show that CRP enrollment is more common among larger operations, indicating that current program structures are more easily utilized by farms with greater land and resource capacity.

By aligning CRP goals with broader agricultural and environmental policies, the program can further address the diverse needs, motivations, and barriers of farmers, ensuring its relevance and effectiveness in the evolving agroecological landscape.

Understanding the factors associated with farmers’ decisions to enroll in CRP can ensure its continued success. Our findings suggest that slope steepness, a proxy ecological variable for low land capability for crop production, was the primary decision factor for the Iowa farmers in our sample. This result underscores CRP’s effectiveness in meeting Congress’s goals, as established through the Farm Bill. Technical assistance from non-governmental sources and the availability of additional financial incentives appear to further encourage enrollment. By understanding these influencing factors, the U.S. Department of Agriculture can continue to promote sustainable land management, enhance environmental health, and support agricultural communities through CRP. Such a comprehensive approach could help stabilize and potentially even increase CRP enrollment to achieve its Congressionally established cap, ensuring the program’s long-term sustainability and expanding its ability to deliver substantial environmental benefits.

Additional research is needed to broaden the geographic and programmatic scope of our findings. A nationwide survey that directly probes farmers’ perceived motivators and barriers to enrolling in each major CRP program, General, Continuous, and Grassland, and to adopting specific practices such as filter strips, pollinator plantings, prairie strips, and wetland restorations, could inform deployment and improve estimates of the cost-effectiveness of achieving conservation outcomes. By oversampling small, tenant-operated, and historically underserved farms and pairing survey responses with FSA enrollment records and geospatial data, research could reveal whether the drivers identified in Iowa (e.g., slope, watershed-group engagement, navigator assistance) hold across regions, program variants, and practice types. Multilevel modeling would clarify how contractual features, information channels, and risk perceptions shape CRP enrollment by accounting for the fact that decisions are nested within counties, conservation districts, or watersheds where rental rates, advisory capacity, and market conditions differ. Now and in the future, such knowledge can provide a robust evidence base to support crucial U.S. Farm Bill legislation.

### Limitations

Several limitations should be considered when interpreting these results. First, the analysis is based on observational survey data and therefore identifies associations rather than causal effects. Although variables such as technical assistance, cost-share receipt, public information sources, and watershed group involvement were positively associated with CRP enrollment, these relationships should not be interpreted as evidence that expanding these mechanisms would necessarily cause higher enrollment. Farmers who seek technical assistance or participate in watershed groups may already differ from other farmers in conservation orientation, land characteristics, or program familiarity. Second, the study is based on Iowa farmers operating at least 60 hectares and is most directly generalizable to row-crop producers in similar Corn Belt contexts. Generalization to other regions, smaller farms, historically underserved producers, or different CRP subprograms should be made cautiously. Third, the dependent variable captures reported CRP participation but does not distinguish between General CRP, Continuous CRP, and Grassland CRP, which may involve different eligibility rules, incentives, and participation motivations. Future research should combine farmer surveys with administrative enrollment records, geospatial measures, and experimental or quasi-experimental designs to evaluate whether specific policy interventions causally affect CRP enrollment.

## Supplementary information

Supplementary information

## Data Availability

The data and materials used in this research will be available upon reasonable request from the corresponding author.
