# Supplementary material for: Factors Associated with Farmers’ Participation in the U.S. Conservation Reserve Program (CRP)
Source: Environ Manage. 2026 Aug 24;76(9):285. doi: 10.1007/s00267-026-02600-3 (PMC13503456; doi:10.1007/s00267-026-02600-3)
Supplement: Supplementary file 1 [file 267_2026_2600_MOESM1_ESM.docx]

**Factors Associated with Farmers’ Participation in the U.S. Conservation Reserve Program (CRP)**

SP1: Factor score and reliability tests for farmers’ attitudes, awareness towards the nutrient reduction strategy, and perceived agronomic and economic capacity to implement conservation practices. Data from the Iowa Nutrient Reduction Strategy Survey, 2015-2019.

| **Survey Items** | **Factor Score** | **CARC*** | **KMO*** | **Bartlett's Test** |
| --- | --- | --- | --- | --- |
| **Attitudes** |  | 0.731 | 0.759 | 0 |
| I would like to improve conservation practices on the land I farm to help meet the Nutrient Reduction Strategy’s goals | 0.746 |  |  |  |
| Helping to meet the Nutrient Reduction Strategy’s goals is a high priority for me | 0.751 |  |  |  |
| I would be willing to have someone help me evaluate how my farm operation is doing in terms of keeping nutrients out of waterways | 0.769 |  |  |  |
| Iowa farmers should do more to reduce nutrients and sediment runoff into waterways | 0.714 |  |  |  |
| **Perceived economic capacity (Cap.Econ)** |  | 0.777 | 0.7 | 0 |
| I can’t afford to implement more conservation practices | 0.803 |  |  |  |
| Many farmers don’t have the economic resources to adopt sufficient conservation practices | 0.799 |  |  |  |
| There is not enough cost-share and other support available from government agencies | 0.726 |  |  |  |
| Pressure to make profit margins makes it difficult to afford conservation practices | 0.688 |  |  |  |
| **Perceived agronomic capacity (Cap.Agron)** |  | 0.787 | 0.609 | 0 |
| Nutrient loss is difficult to avoid in corn-soybean production systems | 0.767 |  |  |  |
| Nutrient loss is difficult to avoid in tile-drained fields | 0.756 |  |  |  |
| Many conservation practices have negative impacts on yields | 0.595 |  |  |  |

SP2: Factor scores and reliability tests for various agricultural stakeholders influencing farmers' decision-making about nutrient management practices and strategies. Data from the Iowa Nutrient Reduction Strategy Survey, 2015-2019.

| **Survey Items** | **Factor Score** | **CARC*** | **KMO*** | **Bartlett's Test** |
| --- | --- | --- | --- | --- |
| **Public sector (Infl.Pub)** |  | 0.854 | 0.816 | 0 |
| NRCS or County Soil and Water Conservation District | 0.834 |  |  |  |
| Iowa Department of Agriculture and Land Stewardship | 0.821 |  |  |  |
| Iowa Water Quality Initiative (WQI) | 0.754 |  |  |  |
| Iowa State University Extension (e.g., field days, workshops, publications, videos) | 0.753 |  |  |  |
| **Private sector (Infl. Priv)** |  | 0.767 | 0.765 | 0 |
| Seed company | 0.826 |  |  |  |
| Local agricultural retailer (e.g., fertilizer, agricultural chemical dealer, co-op) | 0.802 |  |  |  |
| Custom operator/applicator | 0.753 |  |  |  |
| Independent/private crop adviser/agronomist | 0.882 |  |  |  |
| **On-farm research groups (Infl.On-farm)** |  | 0.821 | 0.794 | 0 |
| Practical Farmers of Iowa | 0.848 |  |  |  |
| Iowa Learning Farms | 0.821 |  |  |  |
| Iowa Soybean Association | 0.778 |  |  |  |
| **Family & Landlords (Infl.Fam)** |  | 0.766 | 0.753 | 0 |
| Family members | 0.837 |  |  |  |
| Other farmers | 0.683 |  |  |  |
| Landlord/farm management firm | 0.743 |  |  |  |
